# Assessment of Thermal Transitions by Dynamic Mechanical Analysis (DMA) Using a Novel Disposable Powder Holder

**DOI:** 10.3390/pharmaceutics2020078

**Published:** 2010-03-24

**Authors:** Mohamad G. Abiad, Osvaldo H. Campanella, M. Teresa Carvajal

**Affiliations:** 1 Department of Agricultural and Biological Engineering and Whistler Carbohydrate Research Center, Purdue University, 225 S. University St., West Lafayette, IN 47907, USA; E-Mails: ma192@aub.edu.lb (M.G.A.); campa@purdue.edu (O.H.C.); 2 Department of Industrial and Physical Pharmacy, Purdue University, West Lafayette, IN 47907, USA.

**Keywords:** amorphous powders, glass transition, dynamic mechanical analysis (DMA), powder holder, HPMC, polyethylene oxide, Felodipine

## Abstract

Foods and pharmaceuticals materials are exposed to various environmental conditions during processing and while in storage; therefore, stability and quality are key attributes of concern. The properties of foods and pharmaceutical materials that define their quality are affected by conditions such as temperature, humidity and time. Glass transition is considered a key material property to understand how these external conditions affect the stability and quality of foods and pharmaceuticals. Thus, investigating the thermo-mechanical properties of these materials as well as characterizing the glass transition temperature have a great interest not only in the food industry, but also extend to the pharmaceutical and polymer industries. The aim of this study was to design and test a new disposable powder holder that allows the use of a dynamic mechanical analysis (DMA) instrument to test and characterize loose powder samples. The disposable aluminum powder holder was designed and constructed to be used in the single cantilever configuration on a TA Instruments RSA III DMA. Three different powder samples – Felodipine, polyethylene-oxide (MW 900 kDa) and HPMC (E4M) – were used for validation. The use of this powder holder allows the detection of different thermal changes of powder samples without compacting and when large sample size is necessary for detection and/or interpretation.

## 1. Introduction

Glass transition and melting temperatures along with other thermal changes have been investigated to characterize the solid state of different foods, pharmaceuticals and polymers. Such transitions can be easily detected because they are associated with drastic changes in the viscoelastic properties of the tested material [1]. Thermo-mechanical properties of materials are typically measured by techniques that can assess changes in either mechanical or thermal properties of the material when subjected to controlled temperature histories. Changes in mechanical properties can be monitored using dynamic mechanical thermal analyses (DMTA) also called dynamic mechanical analysis (DMA) whereas changes in the material thermal properties are assessed by differential scanning calorimetry (DSC) or temperature modulated differential scanning calorimetry (MDSC). These methods yield important information on the molecular order and transitions of the tested materials, which are generally temperature dependent. 

DMA is a frequency response analysis that uses a constant, non-destructive oscillatory strain (or stress) at selected frequencies and temperatures while recording the resulting stress (or strain) response of the sample material [2]. The DMA is used to measure glass transition (T_g_) and viscoelastic properties of polymeric amorphous materials. Under tension/compression deformations the measured viscous component is referred to as the loss modulus (E”), while the measured elastic component is referred to as the storage modulus (E’). The ratio of the loss modulus to the storage modulus is referred to as the loss tangent (E”/E’), or tan delta [3]. In addition, a complex dynamic modulus, denoted by E*, is calculated as the magnitude of a complex number which is defined by a real elastic part (E’) and a complex viscous component (E’’); E* = *E'* + *i · E"*. Often, the glass transition, T_g_, of the sample is located as the temperature where tan delta is maximum, a value that usually agrees with the inflection point of the complex dynamic modulus when it is plotted as a function of temperature. However, materials do not have a single glass transition and T_g_ is defined more as the range of temperatures in which the material undergoes drastic changes in its thermo-mechanical properties [4]. Under the test conditions the material was behaving as a viscoelastic solid with temperature varying elastic and viscous moduli. In standard DMA measurements and for the sake of simplicity these moduli are often combined to obtain the complex dynamic modulus E*, from which changes with temperature are monitored. Upon heating, the sample may reach the glass transition temperature range (a material characteristic property) at which the glassy state transforms into a state known as rubbery. At the rubbery state the polymer molecules forming the material can move freely. Although the rubbery state of a material is reached when their forming polymers have more freedom to move, the material viscosity is still extremely high and is not practical for the material processing. In the pharmaceutical industry the use of plasticizers is a common practice to make rubbery materials become flowable. Thus, it is important to differentiate the use of the word rubbery as may have different connotation in the pharmaceutical and food science areas. In both cases rubbery indicate that the material exists in a more movable state, but true flowable materials can be obtained by the use of suitable plasticizers and in many instances that state is recognized as rubbery state. The glass transition temperature range of a sample is dependent upon the composition and compatibility of the components in its amorphous matrix [5,6].

Differential scanning calorimetry (DSC) is another technique utilized to determine glass transition of materials. It involves the simultaneous application of a heat flow to the sample and a reference while measuring the differential heat flow between the sample and a reference. DSC is typically used to detect and measure the melting temperatures of polymers and biomaterials through measurement of enthalpic changes undergone by these materials during phase transitions produced by changes in temperature. The heat (enthalpy) needed for melting (J/g), as well as, the onset, final, and conclusion melting temperatures are typically measured with a DSC instrument. In a conventional DSC, the temperature is increased linearly as a function of time. Usually different molecular segmental motions accompany the glass transition temperature and thus result in small relaxation endotherms [7,8,9,10]. In some cases, the glass transition is hard to detect using the conventional DSC methodology. Temperature modulated DSC is an extension of DSC in which a sine wave modulation is applied to the standard heating rate. This modulation allows the subsequent measurement of the reversing and non-reversing components of the heat flow response [7,8,11,12]. The temperature modulated DSC and conventional DSC are used for detecting the range of T_g_ values which is characterized by a change in the heat capacity of the sample, *i.e.,* the slope of the heat flow curve while the sample passes through its glass transition. 

Even though both DMA and DSC/MDSC methods reveal similar information, the two instruments vary greatly not only in their analyses and sample preparation, but also in their sensitivity. Temperature transitions are more detectable by DMA than by DSC as mechanical changes are more dramatic than changes in the heat capacity. This is because the DMA is able to detect short range motion before the glass transition range is attained and thus identify the onset of main chain motion [13]. Measurements on materials with low moisture contents also present problems when tested by DSC due to indistinguishable transitions and limited accuracy. DSC has also shown to be limited in its use to measure the Tg of certain bio-materials, such as some flours and starches [14,15]. This is attributed to the fact that starch if formed by both amorphous (in where a T_g_ range is observed) and crystalline (in where melting is observed) zones of the starch granule making the T_g_ thermal events difficult to detect specially when those occur close to the melting point [16].

For DMA analysis, the first step is to prepare tablets loaded and pressed under high pressure (5000 lbs or more) to avoid the fracture of the samples during the measurements. That raises questions as to how the sample material is affected due to sample preparation. An alternative testing setup is a DMA powder cell which was designed by TA Instruments to replace powder compaction in an attempt to introduce a testing procedure that will allow the characterization of incompressible powders. The new alternative was tested by Mahlin *et al.* [17] and Abiad [18]. The powder cell can be attached to the dual cantilever clamp on the DMA allowing the characterization of the transitions in powder material as temperature changes by observing the peaks changes in the calculated dynamic complex moduli, E*, also known as apparent moduli. Since the geometry is not well defined and the material is in a powdery state these calculated moduli are only indicative of the elastic and viscous nature of the sample but they do not provide a fundamental measurement. Thus, the magnitudes of the storage modulus and the loss modulus measured using the powder clamp are only qualitative and do not represent the true moduli of the powder; as a result, these moduli are defined as apparent moduli. The use of the powder clamp, however, helps to prevent changes in the active pharmaceutical ingredients (API) structure resulting from the compression of amorphous samples into tablets. 

**Figure 1:**
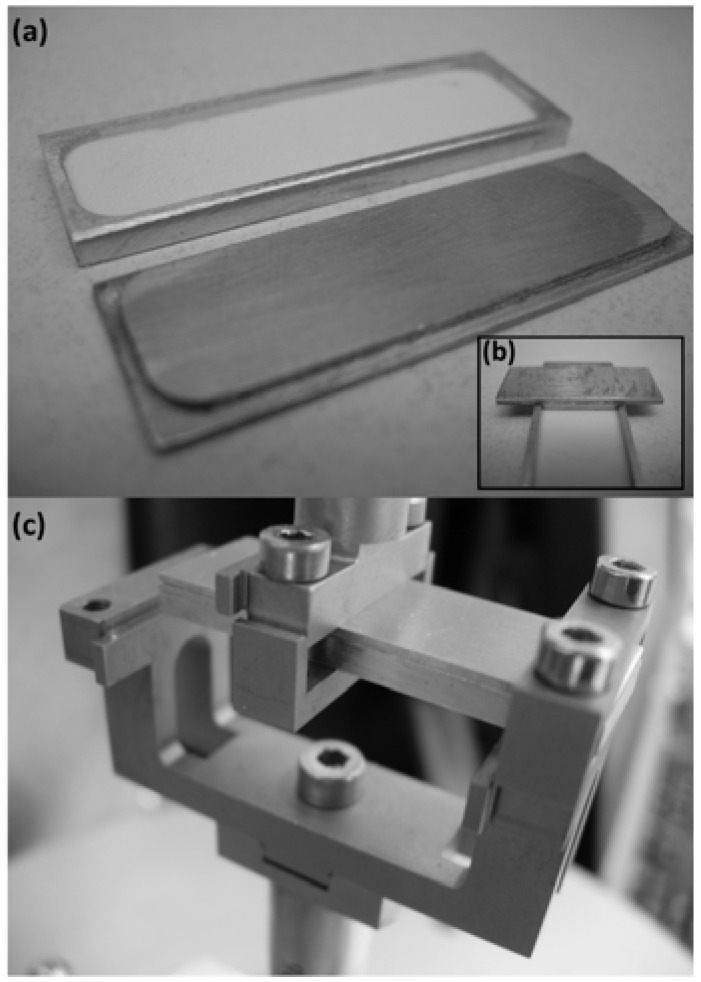
**(a)** Novel disposable powder holder, **(b)** Sample spreading tool to ensure that sample is evenly spread and **(c)** Powder cell attached to the DMA RSA III (TA Instruments) in single cantilever configuration.

In most cases, after the testing is completed it is difficult to remove the lid from the holder due to the fact that the powder after melting acts as glue [17]. Consequently, in this work a new disposable powder sample holder is proposed for use with the dynamic mechanical analysis (DMA) instrument. The new disposable powder cell can be attached to the DMA in a single cantilever configuration (Figure 1). Contrary to the previously suggested cell [17], the novel design eliminates the use of any diluents and abolishes the effect of clamping pressure on the results collected. 

## 2. Experimental Section

### 2.1. Materials

The powders used for this study include hydroxypropyl methyl cellulose (HPMC) E4M, polyethylene-oxide (PEO) – molecular weight 900,000 Da and Felodipine. HPMC-Methocel E4M was kindly donated by The Dow Chemical Company (Midland, MI) batch number WF09012N12. Felodipine was obtained from Astra Zeneca (Mölndal, Sweden) batch number 300051-01 whereas the polyethylene-oxide sample was purchased from Sigma-Aldrich (St. Louis, MO, USA) with batch number 16901MB.

The properties of the active pharmaceutical ingredient, Felodipine, were investigated in the crystalline and amorphous states, the latter obtained by melt-quench cooling of the samples. The melt quenching was done by heating the drug samples to a temperature slightly above their melting point and held for three minutes. The heated melt was then dropped in liquid nitrogen for 10 minutes until the entire sample became solid again. The quenched solids were then crushed using a mortar and pestle and then stored in a Drierite® desiccator – relative humidity ~0% – and kept at -20 ºC. All testing on the active pharmaceutical ingredient was done within 24 hours of sample preparation to avoid re-crystallization of the amorphous powders as much as possible. As for the polyethylene-oxide and the HPMC powders were used as is without any treatment. The values of the glass transition (T_g_) and the melting temperature (T_m_) of the material used in this study as available in literature are presented in Table 1 [19,20,21,22].

**Table 1:** Glass transition (T_g_), re-crystallization and melting temperatures (T_m_) of the powders used in this study [19,20,21,22].

Pharmaceutical Drugs/Additives	Glass Transition Temperature (ºC) T_g_	Re-crystallization Temperature (ºC)	Melting Temperature (ºC) T_m_
HPMC	162		
PEO (MW 900,000 Da)	–		65–70
Felodipine	~43	~98	140–145

### 2.2. Instrumentation

#### 2.2.1. Powder sample holder

The new powder sample holder is designed to hold loose powder eliminating previous sample preparation steps such as tablet forming, thus allowing the direct testing of incompressible powders. The sample holder is an aluminum rectangular container with inner dimensions of 26 × 11 ×1 mm and a lid (Figure 1a). The powder samples can be evenly spread in the container using a novel tool (Figure 1b) and then covered with the lid. The lid is designed in a way that it can also be used to control humidity within the sample using grease around its lips. The cell is then loaded onto the clamped tool using the single cantilever bending geometry (Figure 1c).

#### 2.2.2. Dynamic mechanical analysis (DMA)

A dynamic mechanical analysis (DMA) performed in the RSA III instrument from TA Instruments (New Castle, DE, USA) was used in this study to measure the glass transition temperature, T_g_, and the melting temperatures, T_m_, of the various powder samples. Approximately 100–120 mg of the powder samples were placed in the powder holder and spread evenly in the holder using the special tool. The cell was then covered by the designed lid and attached to the clamped three point bending tool on the DMA apparatus in the single cantilever configuration. The powder samples were then heated at rate of 5 ºC/min with an applied frequency of 1 Hz and a strain level of 0.01%. Normalized complex modulus (E*) was plotted *versus* temperature to determine the glass transition or the melting temperatures. The normalized complex modulus was obtained by taking the ratio of the complex moduli within a sample set to the value of the maximum modulus in the same data set.

The range of temperatures used for the testing depended on the particular sample. The amorphous and crystalline Felodipine samples were heated from 25 ºC up to 70 ºC and 158 ºC, respectively; HPMC was heated from 40 ºC to 200 ºC whereas polyethylene oxide was heated from 30 ºC to 85 ºC. The temperature was ramped at 5 ºC/min with an applied frequency of 1 Hz and a strain level of 0.01% For analysis, a normalized complex modulus (E*) was plotted *versus* temperature to determine Tg values. The normalized complex modulus was obtained as the ratio of the complex modulus at a given temperature and the value of the maximum modulus obtained in the same data set. That maximum value coincides with the complex modulus of the sample at the glassy state. Results were compared with those obtained from DSC and MDSC testing. Sample testing was done in triplicates.

#### 2.2.3. Differential Scanning Calorimetry (DSC) and Modulated Temperature DSC (MDSC)

Approximately 4 ± 1 mg of the Felodipine and PEO samples were placed in hermetically sealed aluminum pans and scanned at 5 ºC/min in a differential scanning calorimeter (DSC) model Q2000 (TA Instruments, New Castle, DE, USA) under a nitrogen gas flow of 50 cc/min. As discussed the temperature range varied from sample to sample. Similarly to DMA testing scans for the amorphous and crystalline samples of the Felodipine were from 25 ºC to 70 ºC and 158 ºC, respectively; polyethylene oxide was from 30 ºC to 85 ºC. For the HPMC E4M samples pin-holed hermetically sealed aluminum pans were used in temperature modulated mode. The underlying heating rate for the HPMC samples was set to 3 ºC/min from 40 ºC to 240 ºC with modulation amplitude of 0.32 ºC and a period of 40 s. For analysis, melting endotherms and T_g_ values were determined from the change of the sample heat capacity. All runs were done in triplicates and averages are reported.

## 3. Results and Discussion

The thermal and thermo-mechanical properties utilized to determine the glass transition and melting temperatures of the powder samples were assessed using DMA and DSC/MDSC methodologies. The plots for the DMA show the normalized dynamic complex modulus as a function of temperature. The glass transition as well as the melting temperatures are reported as the inflection point at the step change in the E* curve. For the DSC measurements, the glass transition temperature is observed as a sudden change in heat capacity or a shift in the heat flow curve, whereas the melting temperature is reported as the endothermic peak on the heat flow – temperature graph. All results were averaged and reported to be used for the validation of the new proposed disposable powder holder.

### 3.1. Glass Transition Temperature of HPMC E4M

**Figure 2:**
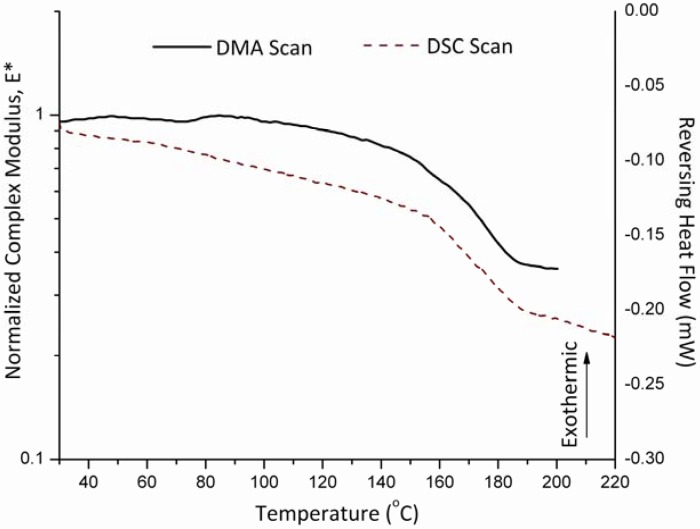
Mechanical response of HPMC (E4M) sample in the powder sample holder as compared to a DSC scan showing the glass transition temperature occurring around 175 ºC.

The estimation of the glass transition temperature for the HPMC samples from MDSC experiments was obtained from the midpoint of the typical heat capacity change that was observed in the reversing heat capacity graphs. The Tg value for this sample obtained by MDSC was 173.0 ± 6.4 ºC. This result is in agreement with the one determined by the DMA which was 175.0 ± 4.4 ºC. These results are illustrated Figure 2. 

**Figure 3:**
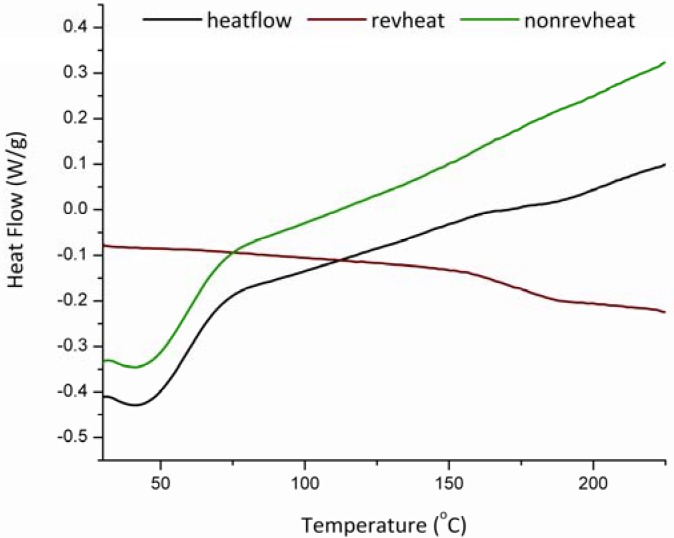
DSC scan for HPMC (E4M) the line in black shows the total heat flow of the sample, the red line shows the reversing heat flow indicating the glass transition temperature whereas the green line shows the non-reversing heat flow.

It is clearly identified from the DMA results that the glass transition temperature is accompanied by a significant loss of the normalized complex modulus, E*. On the other hand, conventional DSC measurements for HPMC (E4M) do not show a clear location of the glass transition temperature as Figure 3 illustrates when observing the total heat flow. However, resorting to the MDSC option and plotting the reversing and non-reversing heat flows, the MDSC plots then are able to locate the change in heat flow corresponding to the glass transition phenomenon. Consequently, this gives an advantage for the use of DMA over that of conventional DSC especially when strong glasses such as HPMC are considered.

### 3.2. Melting of Polyethylene Oxide (PEO)

**Figure 4:**
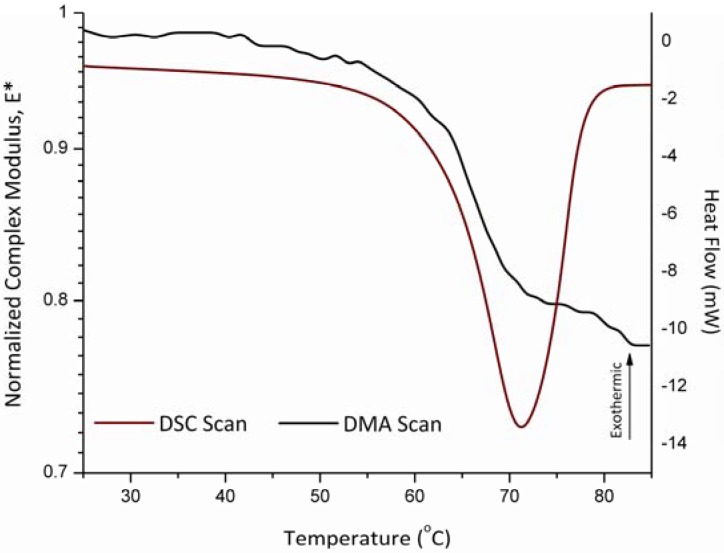
Mechanical response of polyethylene-oxide 900,000 Da sample in the powder sample holder as compared to a DSC scan showing the melting temperature occurring around 63 ºC.

The melting temperature of polyethylene oxide with molecular weight of 900 kDa, as measured using DSC was 63.0 ± 0.7 ºC. Using the powder sample holder on the DMA in the single cantilever configuration resulted in a value of 65.0 ± 1.7 ºC for the melting temperature. These two measurements are in agreement. Figure 4 shows both the DSC scans plotted as heat flow *versus* temperature while the DMA scans are plotted as the normalized dynamic complex modulus as a function of temperature. 

### 3.3. Glass Transition and Melting Temperatures of Crystalline Felodipine

**Figure 5:**
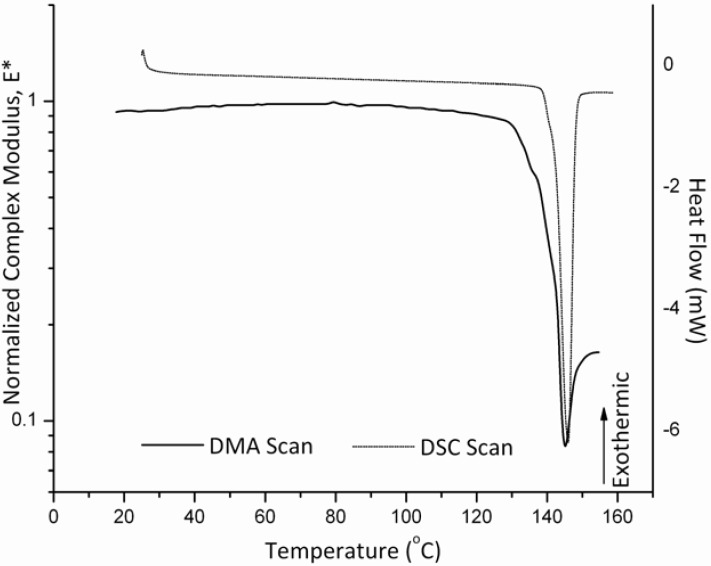
Mechanical response of crystalline Felodipine sample in the powder sample holder as compared to a DSC scan showing the melting temperature occurring around 143 ºC.

**Figure 6:**
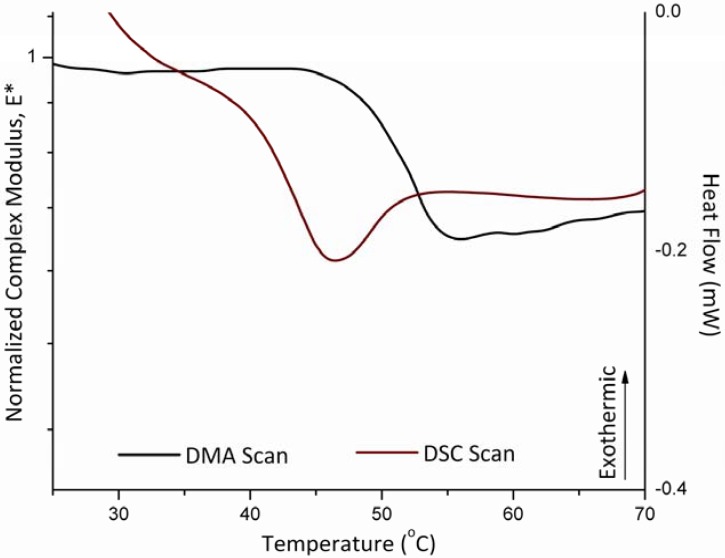
Mechanical response of amorphous Felodipine sample in the powder sample holder as compared to a DSC scan showing the glass transition temperature occurring around 46 °C.

The Felodipine powder samples were scanned to measure both the glass transition of the amorphous sate and the melting temperature of the crystalline powder. The DSC results illustrated in Figure 5 and Figure 6 show a glass transition temperature of 45.0 ± 2.7 ºC and a melting temperature of 144 ± 1.4 ºC. On the other hand, the analysis of the results obtained by the DMA show a glass transition for the Felodipine at 50.0 ± 0.7 ºC a melting temperature of 142.0 ± 1.4 ºC (Figure 5 and Figure 6). Both results determined using the DMA are in agreement with those obtained from the conventional DSC method.

Figure 4 through Figure 6 illustrate the differences between thermodynamic and kinetic events. Melting is a thermodynamic (equilibrium) transition characteristics of a crystalline material. In other words, at a given pressure and for a given material the measured melting temperature it is a property of the material, this should be independently of the instrumental technique or experimental conditions. Consequently, the DMA and DSC methods show very close agreement during the measurements as to the location of the melting events in crystalline material (Figure 4 and Figure 5). Conversely, the glass transition is a kinetically controlled event phenomenon whose observed position (and shape) reflects the properties of the material in response to specific experimental conditions of the measurement [23]. In this sense, each instrumental technique reveals a different aspect of the glass transition. Figure 6 shows that the calorimetric glass transition is observed earlier than its mechanical counterpart. Although both tests were performed using the same heating rate, Figure 6 indicates that the shift in heat capacity becomes clearly detectable before the corresponding change in the sample mechanical properties that accompany the glass transition. Heating of a glassy material through the glass transition, results in a reduction in the size of the cooperative rearranging regions (CRR) [24]. Figure 6 suggests that a threshold in the reduction of the CRR size may be needed before the effect is clearly reflected on the resulting material mechanical properties. It is noteworthy that the endpoints of the calorimetric and mechanical glass transition are considerably closer than their respective onsets. 

### 3.4. Effect of Frequency and Heating Rate on HPMC E4M Results

**Figure 7:**
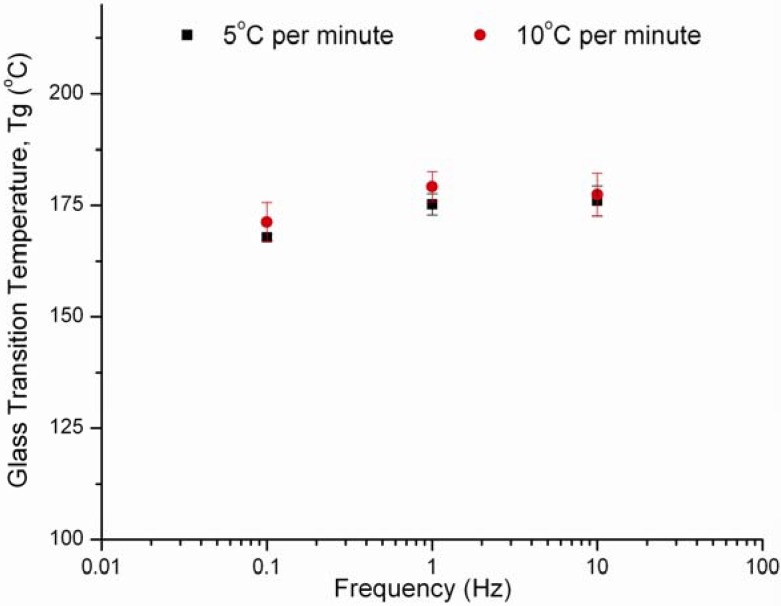
Plot showing Tg as a function of frequency at the heating rates of 5 and 10 ºC per minute.

In addition to the experiments conducted to determine the glass transition and melting temperatures of the previously discussed materials, the effects of heating rate and frequency were investigated. Two heating rates of 5 and 10 ºC per minute were considered along with three frequencies of 0.1, 1 and 10 Hz. Table 2 illustrates glass transition temperatures of HPMC E4M determined at the different conditions. Tukey’s student range test with α = 0.05 was used to analyze the collected data. According to Tukey’s grouping, the data showed no significant difference between the temperatures obtained at the various predefined conditions. Consequently, this shows that in cases where the material is considered to be a strong glass such as HPMC, frequency and heating rates have no significant effect on the glass transition temperature as is the case with other weaker glasses. A plot of the glass transition temperature as a function of frequency at the two different heating rates is illustrated in Figure 7.

**Table 2:** Glass transition temperature of HPMC E4M determined at two different heating rates and various frequencies with the respective statistical analysis including Tukey’s studentized range test with α = 0.05 compared against the various frequencies as well as heating rates.

Heating rate	Frequency Hz	Mean Tg (n = 3) (°C)	Standard Deviation (°C)	Percent Error (%)
**5 °C/min**	0.1	168^a^	0.69	0.41
	1.0	175^a^	2.38	1.36
	10.0	176^a^	3.37	1.92
**10 °C/min**	0.1	171^a^	4.40	2.57
	1.0	179^a^	3.36	1.87
	10.0	177^a^	4.83	2.72

## 4. Conclusions

**Figure 8:**
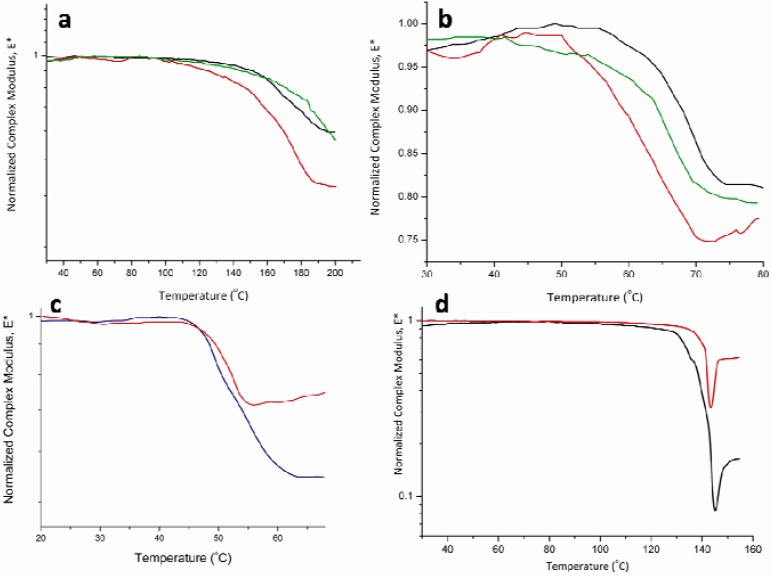
DMA results using the powder cell showing reproducibility for (a) HPMC (E4M), (b) Polyethylene Oxide, (c) Amorphous Felodipine and (d) Crystalline Felodipine.

Based on the results obtained with the various samples tested, it is apparent that the novel powder cell offers a practical, sensitive and reproducible method to measure the glass transition temperatures as well as melting temperatures of various powders using the dynamic mechanical analysis instrument. The testing results are reproducible and that can be seen in the HPMC samples as well as the other API’s and polymer used for the study (Figure 8). In addition to measuring the glass transition temperature of the pure material, this newly developed cell expands the usage of the DMA to include applications on incompressible powders. Since changes in the thermo-mechanical properties of a given material are more dramatic than changes in its heat capacity, this novel powder holder allows the determination of thermal transitions in samples where non-mechanical approaches such as the DSC fail to clearly define such changes. Unlike the previously designed powder holder [17], this novel powder cassette eliminates the use of diluents. In addition, it abolishes the effect of clamping pressure on the obtained results and the data being collected.
